# The difference of human gut microbiome in colorectal cancer with and without metastases

**DOI:** 10.3389/fonc.2022.982744

**Published:** 2022-11-01

**Authors:** Leitao Sun, Zhenzheng Zhu, Xinru Jia, Xiangchang Ying, Binbin Wang, Peipei Wang, Shuo Zhang, Jieru Yu

**Affiliations:** ^1^ Department of Medical Oncology, The First Affiliated Hospital of Zhejiang Chinese Medical University (Zhejiang Provincial Hospital of Chinese Medicine), Hangzhou, Zhejiang, China; ^2^ The First School of Clinical Medicine, Zhejiang Chinese Medical University, Hangzhou, Zhejiang, China; ^3^ The Second Affiliated Hospital of Zhejiang Chinese Medical University (Xinhua Hospital of Zhejiang Province), Hangzhou, Zhejiang, China; ^4^ School of Basic Medical Sciences, Zhejiang Chinese Medical University, Hangzhou, Zhejiang, China

**Keywords:** metastasis, metastatic colorectal cancer, gut microbiota, biomarkers, colorectal cancer

## Abstract

Metastasis of colorectal cancer is deemed to be closely related to the changes in the human gut microbiome. The purpose of our study is to distinguish the differences in gut microbiota between colorectal cancer with and without metastases. Firstly, this study recruited colorectal cancer patients who met the established inclusion and exclusion criteria in the Oncology Department of Zhejiang Hospital of Traditional Chinese Medicine from February 2019 to June 2019. Fresh stool samples from healthy volunteers, non-metastatic patients, and metastatic patients were collected for 16S rRNA gene sequencing, to analyze the diversity and abundance of intestinal microorganisms in each group. The results showed that the microbial composition of the control group was more aplenty than the experimental group, while the difference also happened in the Tumor and the metastases group. At the phylum level, the abundance of *Bacteroidetes* significantly declined in the Tumor and the metastases group, compared with the control group. At the class level, *Bacilli* increased in experimental groups, while its abundance in the Tumor group was significantly higher than that in the metastases group. At the order level, the Tumor group had the highest abundance of *Lactobacillales*, followed by the metastases group and the control group had the lowest abundance. Overall, our study showed that the composition of the flora changed with the occurrence of metastasis in colorectal cancer. Therefore, the analysis of gut microbiota can serve as a supplement biological basis for the diagnosis and treatment of metastatic colorectal cancer which may offer the potential to develop non-invasive diagnostic tests.

## 1 Introduction

Colorectal cancer (CRC) is not only considered the third most common malignancy in the world (1), but also the second most deadly cancer (2). Meanwhile, over recent years, its incidence has continued to rise (3). According to estimation, there will be 3.2 million new cases globally by 2040 (1). About 20% of CRC patients have metastases at diagnosis while another 20% who develop metastases at the time of follow-up need systemic treatment (4). It is named Metastatic Colorectal Cancer (MCRC). Therefore, the prognosis of colorectal cancer remains poor though surgery, despite radiotherapy and chemotherapy has advanced significantly (5).

Gut microbiota (GM) is the largest microbiome in the human body which involves at least 1,000 different species of bacteria and 100 trillion microbes (6). It plays a vital role in maintaining intestinal stability (7) as well as healthy state by eliminating pathogens and establishing intestinal barriers (8).

In recent decades, it has been proven that the dysregulation of intestinal flora forms biofilm, which leads to damage of intestinal barrier function, further enhances intestinal dysregulation, and promotes the development of colon cancer (9). It is worth emphasizing that there is a complicated relationship between CRC and GM (10). Research has already confirmed that the GM diversity of patients with CRC is lower than healthy people (11). As probiotics, butyrate-producer, *Clostridium butyicum*, and lactate-producer like *S. thermophilus* in CRC patients were exhausted (12). Meanwhile, some species of CRC patients’ gut microbiota showed a significant increase. For instance, in cancer tissue, there are more Fusobacterium than adjoining health tissue (13) which will foster tumor proliferation during the development of CRC (14). Meanwhile, the study has shown that gut microbiota could further promote CRC metastasis by interfering with metabolism (15).

**Figure 1:**
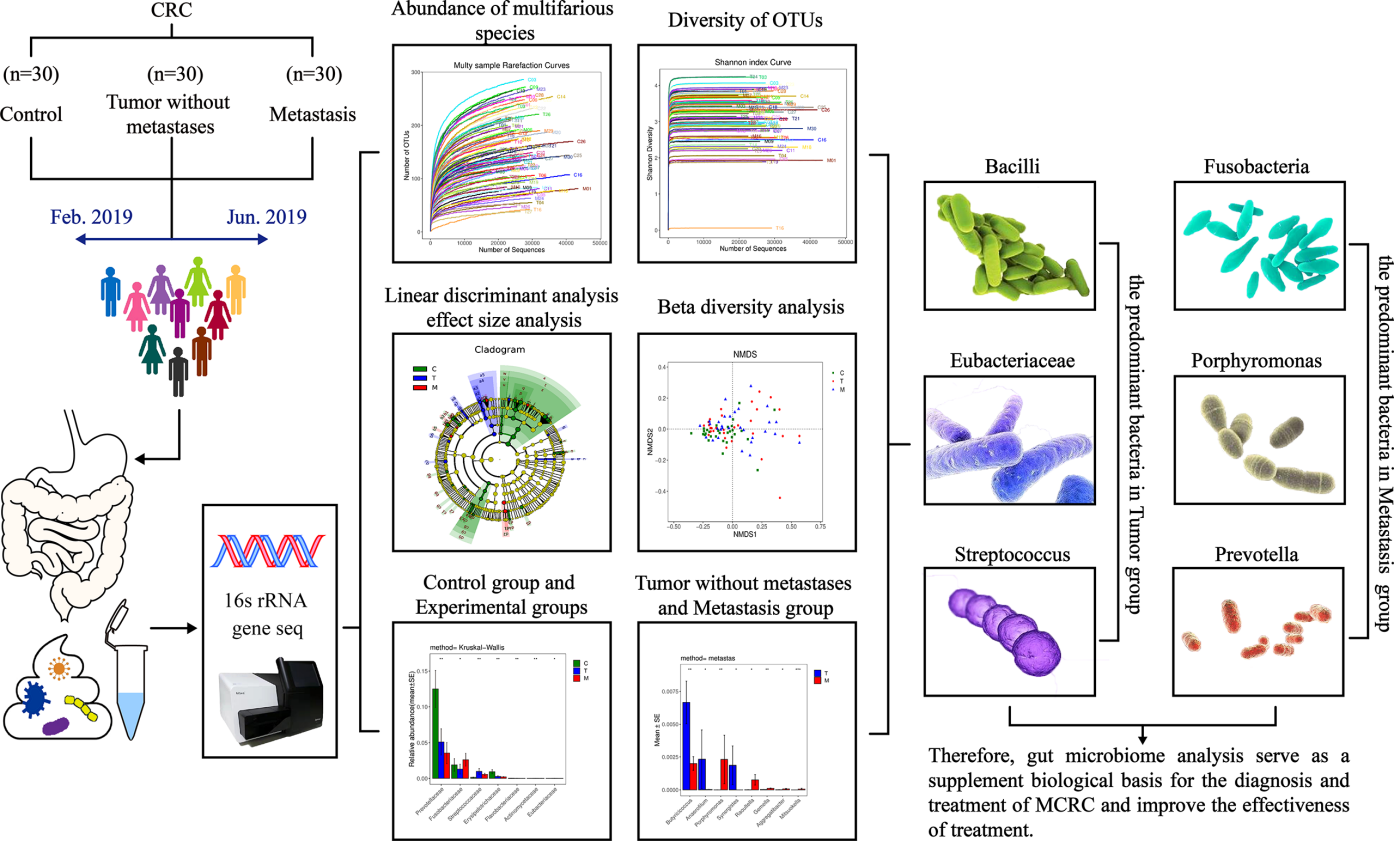
Human gut microbiome in colorectal cancer (CRC) without metastases and metastases.

As the vast majority of the existing studies have focused on the relationship between CRC and gut microbiota, there is a gap in flora in the colorectal cancer metastasis research field. This study intends to distinguish the differences in gut microbiota between colorectal cancer with and without metastases, to supplement the biological basis for the treatment of metastatic colorectal cancer. The design is shown in **Figure 1**.

## 2 Material and methods

### 2.1 Patient recruitment

The collection of samples was carried out in the Oncology Department of Zhejiang Provincial Hospital of Traditional Chinese Medicine between February 2019 and June 2019. The inclusion criteria of the experimental groups were as follows: (a) Individuals who meet the diagnostic criteria and are diagnosed with colorectal cancer by histopathological examination; (b) Individuals with no family history of CRC; (c) People aged between 18 to 75 years old; (d) Individuals who have no diarrhea, vomiting, nausea and other gastrointestinal discomforts during the previous month; (e) Individuals who did not use antibiotics or probiotics in the 4 weeks before taking stool samples. Patients with the following conditions were excluded: (a) Pregnant or breastfeeding women; (b) Individuals with inflammatory, infectious, or other autoimmune diseases; (c) Individuals with fecal discharge through fistulas; (d) Uncooperative people. The research was conducted under the clinical studies rules at home and abroad. All the protocols and procedures of this study were approved by the Zhejiang Province Hospital of TCM Ethics Committee (2020-KL-050-01), and all volunteers signed the informed consent form before participating in the experiment.

### 2.2 Diagnostic criteria

All patients had no history of malignant tumors except for the diagnosis of CRC, which was confirmed by pathology and operation. Case classification and grouping were carried out following the eighth edition American Joint Commission on Cancer (AJCC) TNM staging system (16).

### 2.3 DNA samples test

The stool samples were collected from CRC patients and healthy volunteers in a clean environment, preserved in an aseptic sampling tube at −80°C. A 200 mg fecal sample was weighed into a 2 ml centrifuge tube. Then, the DNA from the stool samples was extracted by QIAamp DNA Fecal Mini Kit (Qiagen, Germany) according to the instructions. The samples were added ASL and incubated in a water bath at 95°C for 5 min. The process of extracting the final DNA was performed in 50 µL AE. The extracted total DNA was tested for concentration, purity, and integrity by 1% agarose gel electrophoresis and NanoDrop2000 ultramicro spectrophotometer, then stored at 4°C for further analysis.

Three groups were set in the experiment, namely the C (Control) group, the T (Tumor without metastases) group, and the M (Metastases) group.

### 2.4 PCR amplification

The hypervariable region of the microbial 16S rRNA gene was amplified by PCR thermocycler. The sequences for the primers were 357F (5’-ACTCCTACGGRAGGCAGCAG-3’) and 806R (5’-GGACTACHVGGGTWTCTAAT-3’). The concrete scheme is as follows: 3 min at 95°C, then 29 cycles of 30 s at 95°C, 30 s at 55°C and 45 s at 72°C, followed by 5 min at 72°C. The PCR reaction was repeated in three times. During the pre-experiment, ten samples were selected randomly to produce the lowest cycle number, and the results showed that most of the samples could be scaled up to the appropriate concentration of the product. Formal experiments were then performed using the TransGen AP221-02, TransStart Fastpfu DNA Polymerase, and 20-μl reaction system. The products of PCR were quantitatively detected with a micro fluorometer based on the results of preliminary sample detection electrophoresis. Afterward, the products were mixed according to the sequencing volume of a single sample.

### 2.5 Bioinformatics analysis

#### 2.5.1 Majorizing sequence

Paired-end DNA was sequenced by Illumina Miseq. The results of the sequencing were extracted according to the barcode tag complete matching method and transformed into images. Raw fastq files were quality-filtered by Trimmomatic and merged by FLASH. The specific process was as follows: In the 50bp window, if the average quality value was lower than 20, the back-end bases were truncated from the window, and the reads below 50bp after quality control were filtered. According to the overlap relationship between PEreads, the paired reads were merged into one sequence, and the minimum overlap length was 10bp. The maximum mismatch ratio allowed in the overlap region of the spliced sequence was 0.2. Mothur V.1.39.5. (Parameter settings: maxambig=0, maxhomop=8, minlength=200, maxlength=485) was used to filter out the singletons in the spliced long reads (corresponding to a sequence with only one read) to obtain data for subsequent clustering OTUs.

#### 2.5.2 OTU analysis

Operational Taxonomy Unit (OTU) is a set of operational definitions used to classify a certain taxonomic unit including domain, kingdom, phylum, class, order, family, genera, and species, which facilitates the analysis in population genetics or phylogenetic research. Clustering was performed at 97% similarity by UPARSE software to obtain representative sequences of OTUs. Chimeras generated by PCR amplification were then removed from the OTU representative sequences by UCHIME software and golddatabase (v20110519). The abundance of each sample at each OTU was obtained by ussearch_global method. Then, the representative sequences of OTUs were aligned with Silva128, Greengene, and RDP databases by mothur (classify.seqs) software for species annotation. The confidence threshold was 0.6.

#### 2.5.3 Taxonomic analysis

According to the taxonomic information, the community structure was statistically analyzed at the taxonomic levels of phylum, class, order, family, genus, and species, and the number of sequences at different taxonomic levels was counted for each sample respectively.

#### 2.5.4 Rarefaction curve

The majorizing sequence was randomly selected from the OTU sequence with a similarity of 97%. According to the number of corresponding OTU and the number of the selected majorizing sequences, the rarefaction curve was constructed and drawn R software (version 3.6.3).

#### 2.5.5 Alpha diversity analysis

Four indices were used to measure alpha diversity. The Chao index and Ace index were calculated to estimate the number of species in the samples, while the Shannon index and the Simpson index reflected the community diversity. Mothur V.1.39.5. and Qiime were used for Alpha diversity analysis.

### 2.6 Community histogram

Based on the results of the taxonomic analysis, the community structure composition of different classification levels was obtained, and the corresponding diagrams were drawn by the R language tool.

### 2.7 Multi-level species differences analysis

Linear discriminant analysis effect size (LEfSe), a software for discovering high-dimensional biomarkers and revealing genomic features, was used to perform linear discriminant analysis on samples with different grouping conditions in line with the classification composition. Then, communities or species that were significantly affected by the differences in sample division were screened by linear discriminant analysis (LDA). The software used in this experiment is lefse (1.0).

### 2.8 Difference analysis between groups

Differentially Abundant Features can be evaluated by multiple hypothesis testing and false discovery rate (FDR) analysis of rare frequency data based on the obtained OTU or community abundance. In the metastats software, the samples of the general control group and the experimental group were analyzed and compared at each level to find out the different bacterial species with certain differences in bacterial abundance. Differences were considered statistically significant when the P value was< 0.05.

### 2.9 Statistical analysis

The difference in the flora data among the C group, the T group, and the M group were evaluated with statistical software (SPSS 22.0). Data were expressed as mean ± standard deviation ( $\bar{x}\pm \text{s}$ ). The differences between two independent samples were compared by the T-test, while the differences between groups were analyzed by Kruskal -Wallis and Wilcoxon rank-sum test. Differences were considered statistically significant at *p*<0.05, significant at *p*<0.01, and not statistically significant at *p*>0.05.

## 3 Results

### 3.1 General information

This study collected a total of 90 cases, including 30 cases in the C group, 30 cases in the T group, and 30 cases in the M group. There were 47 males and 43 females, aged from 17 to 75 years, with an average of 55.92 ± 10.70 years old. There was no significant difference in any clinical factors such as gender, age, or inflammation location among the individuals. (*p*>0.05) (**Table 1**)

**Table 1:** Characteristics of healthy volunteers and CRC patients.

	C group (n)	T group (n)	M group (n)
Cases	30	30	30
Gender			
Male	11	19	17
Female	19	11	13
Age (mean ± SEM)	52.6 ± 11.2	57.5 ± 9.3	57.3 ± 10.7
Smoking history (Y/N/unknown)		7/23/0	15/15/0
Drinking history (Y/N/unknown)		4/23/3	12/16/2
Duration of disease (years)			
<2		0	0
2-4		21	14
>4		9	16
Tumor location			
Rectum		13	16
Left colon		6	3
Right colon		2	0
Colon, site unknown		9	12
Metastasis site			
Liver			12
Lung			15
Bone			7

### 3.2 Serial data statistics

This study collected a total of 90 cases, including 30 cases in the C group, 30 cases in the T group, and 30 cases in the M group. According to the general principles of systems genetics and population genetics, the fuzzy and repeated base sequences that affected the quality of analysis were eliminated, and 2425308 optimized sequences were accurately obtained.

### 3.3 Sequencing depth and the analysis of sample size

**Figure 2:**
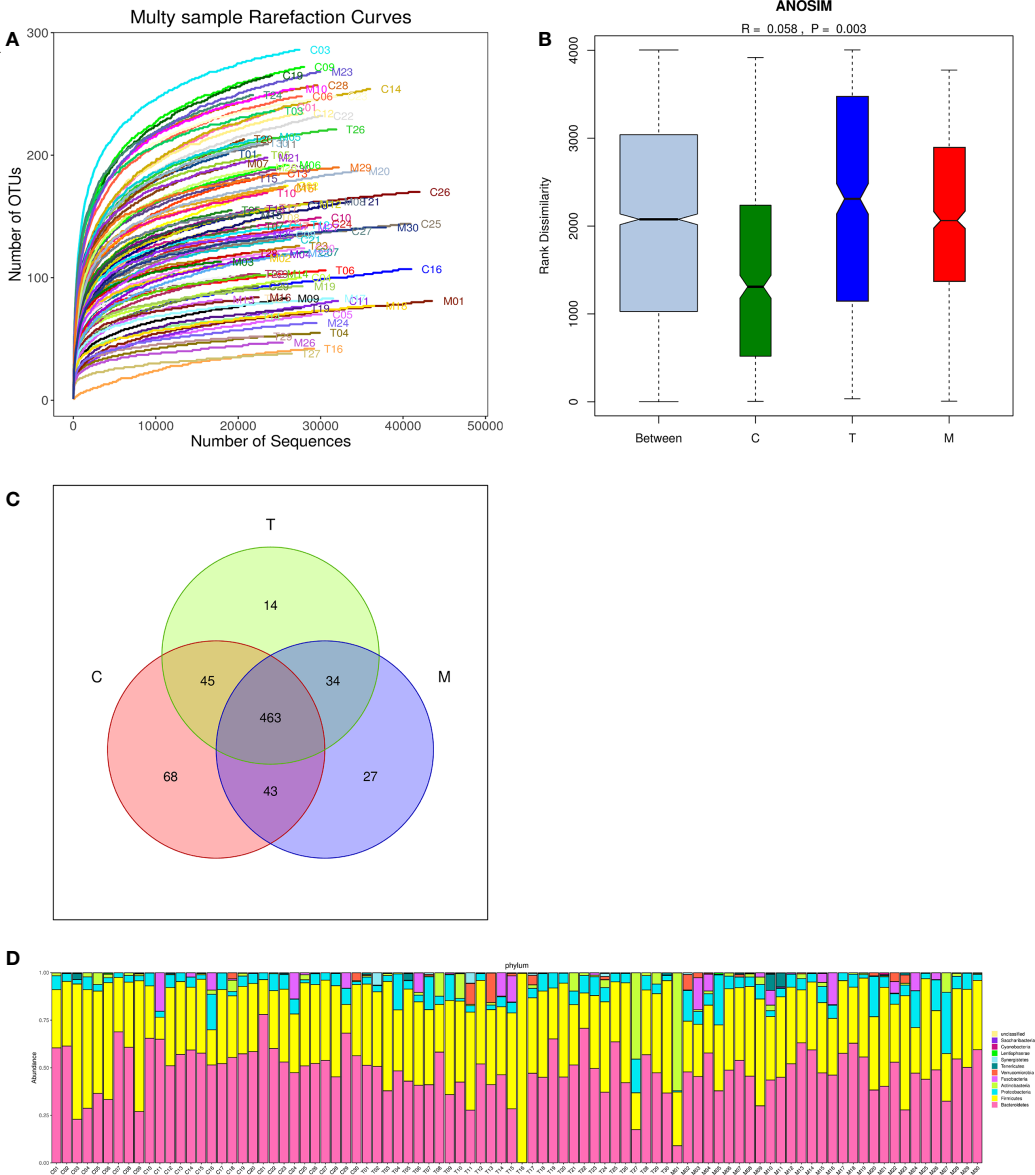
Multiple sparse curves for comparing the abundance of multifarious species **(A)**, Similarity analysis boxplot for identifying the existence of differences between groups **(B)**, Venn diagram for implying the common and specific traits among three groups **(C)**, Histogram of the abundance distribution of species at the phylum level **(D)**.

There were 694 OTUs clustering in the 90 samples in this study, including 199 species, 138 genera, 47 families, 30 orders, 18 classes, 11 phyla, and 1 domain. More data contributed little to the discovery of new OTUs as the curve flattened, suggesting that this study can be conducted with reasonable sample collection and high species richness (**Figure 2A**). Additionally, the similarity analysis indicated that the differences between the groups were more obvious than the difference within the groups (R=0.058, *p*<0.01), declaring the amount of data in this study was feasible to reflect most of the information on the microbiota in each group objectively (**Figure 2B**). Venn diagram (VENN) showed the number of OTUs that were distinct or the same in each set of intestinal tracts. The total number of OTUs in this study was 694, of which the number of OTUs in the general control group was exceedingly higher than that in the experiment groups (619 vs 556,567) (**Figure 2C**). According to the sample community’s structures, the abundance diversity histogram of the top twelve phylum species suggested that *Bacteroidetes*, *Firmicutes*, *Proteobacteria*, *Actinobacteria*, *Fusobacteria*, and *Verrucomicrobia* were the predominant flora in most samples and the proportions of them were different in each group (**Figure 2D**).

### 3.4 Diversity analysis

**Figure 3:**
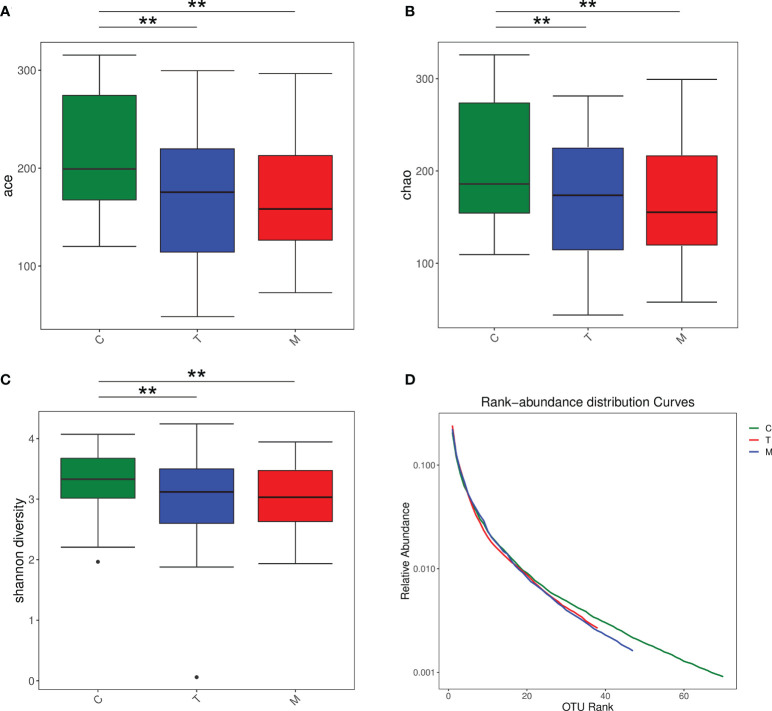
Boxplot of the Kruskal -Wallis test for the Ace index **(A)**, Boxplot of the Kruskal -Wallis test for the Chao index **(B)**, Boxplot of the Kruskal -Wallis test for the Shannon index **(C)**, Rank-abundance distribution curves of the general control group and the experimental groups **(D)**. **p < 0.01.

We performed Kruskal-Wallis test for the Ace, Chao and Shannon index of the three groups and the results showed that the intestinal microbiota diversity of the C group was higher than that of the other two experimental groups (**Figures 3A–C**). Based on the corresponding OTU number and the selected sequence number, rank-abundance distribution curves showed that the species abundance and uniformity of the T and M group were lower than the C group (**Figure 3D**). Besides, due to the problems in sample quality and detection process, sequencing results of T16 were meaningless.

### 3.5 Analysis of species’ differences between groups

#### 3.5.1 Beta diversity analysis based on OTU

**Figure 4:**
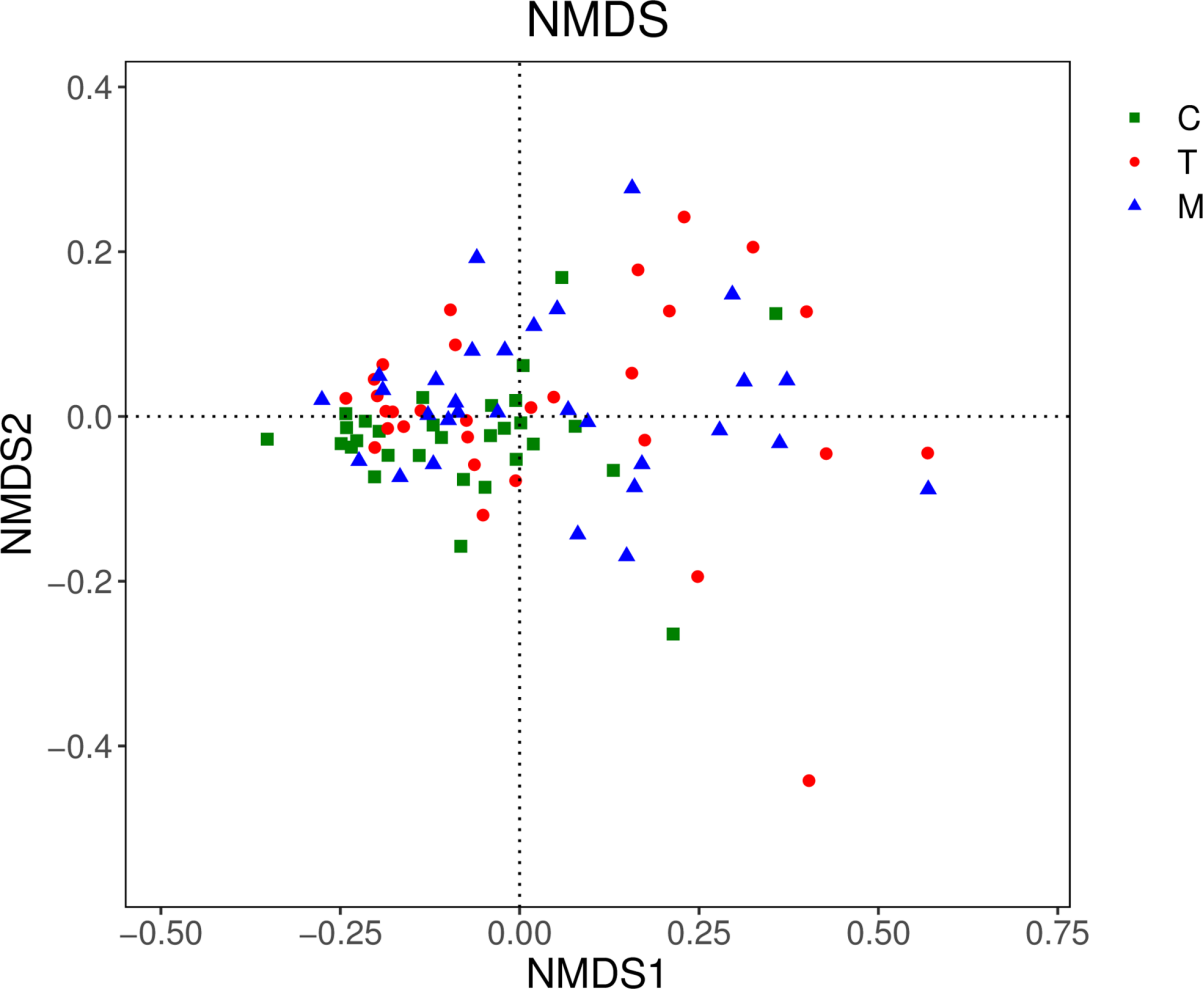
NMDS diagram of the general control group and the experimental groups.

In the NMDS chart, the degree of difference between diverse samples was reflected by the distance between the points. Points represented the 3 groups distributed on the diagram regularly, which indicated that there were significant differences in OTUs types among the three groups. (**Figure 4**).

#### 3.5.2 Discriminant analysis of community differences among LEfSe

**Figure 5:**
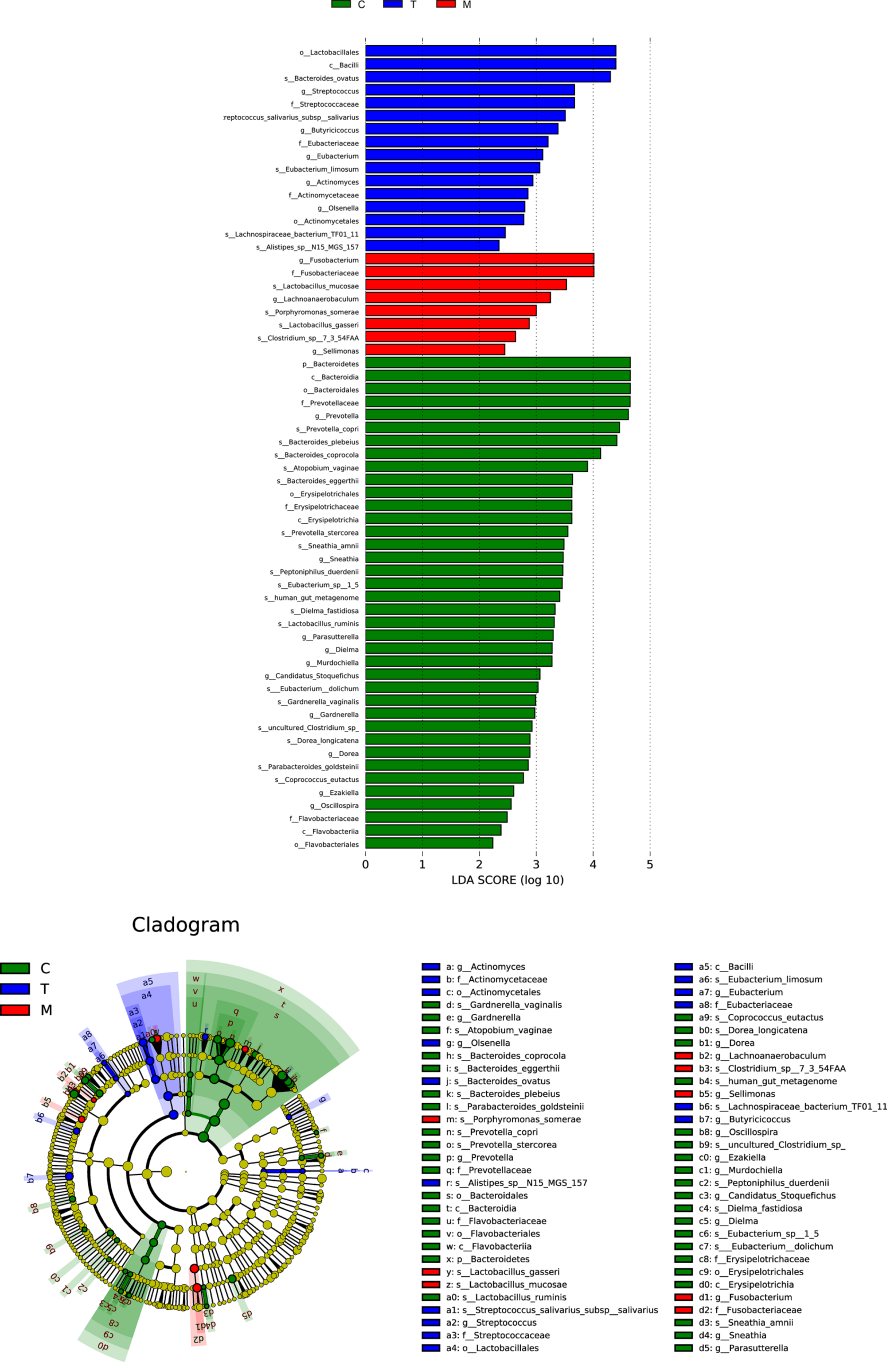
Histogram and cladogram of Linear discriminant analysis effect size (LEfSe) analysis among the general control group and the experimental groups.

The concentric circles from inside to outside are phylum, class, order, and family, and its nodes correspond to specific flora of different classes. Under the LEfSe diagram (**Figure 5**), the result indicated the dominant flora of each group: *Bacteroidetes* on the phylum level in the control group; *Bacilli* on the class level, *Lactobacillales* on the order level, *Eubacteriaceae* on the family level, *Streptococcus* and *Butyricicoccus* on the genus level in the tumor without metastases group; *Fusobacterium* and *Lachnoanaerobaculum* on the genus level, *Porphyromonas_somerae* on the species level in the metastases group.

#### 3.5.3 Nonparametric test based on species information

**Figure 6:**
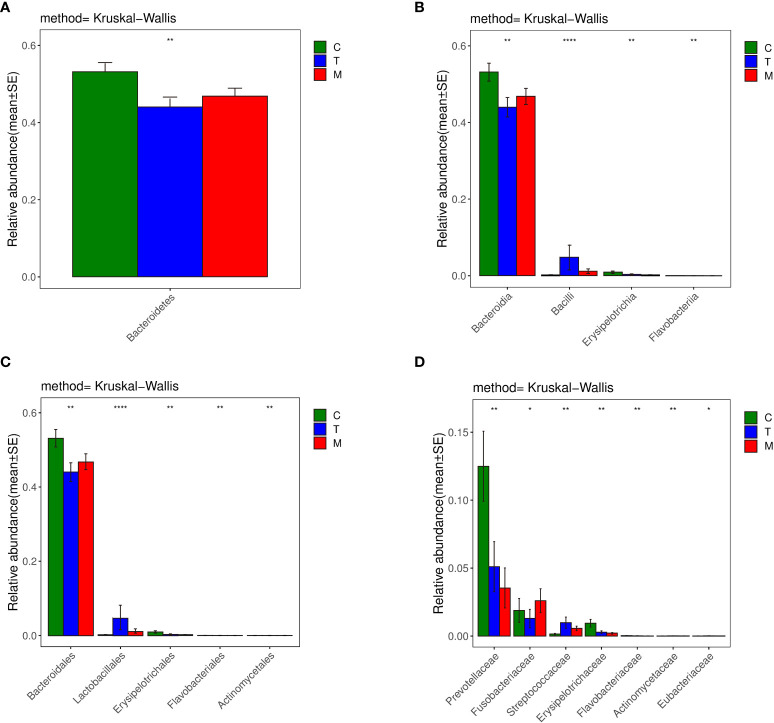
Histograms of differences in the diversity of species analyzed by kruskal_ Wilcox test among three groups at the levels of phylum **(A)**, class **(B)**, order **(C)**, family **(D)**, genus. *p < 0.05, **p < 0.01, ***p < 0.001, ****p < 0.0001.

*Bacteroidetes* accounted for 44.0% and 46.8% of the total amount of bacteria in the T and the M group based on the histogram of phylum (**Figure 6A**), which were lower than 53.2% in the C group (*p*<0.01). So, *Bacteroidetes* was the predominant bacteria in the C group.

Considering the class histograms (**Figure 6B**), *Bacilli* accounted for 4.8% and 1.2% of the total number of bacteria in the T and the M group, which were higher than that in the C group, which accounted for 0.2% (*p*<0.01). Therefore, *Bacilli* was the predominant bacteria in the T and M group. *Erysipelotrichia* accounted for 0.3% and 0.2% of the total amount of bacteria in the T and the M group, which were less abundant than that in the C group, which accounted for 0.9% (*p*<0.01). The abundance of *Flavobacteriia* in the C group was higher than that in the other two groups (0.02% VS 0.01%, 0.003%) (*p*<0.01). Thus, the specific bacteria in gut bacterial composition of the C group were *Erysipelotrichia* and *Flavobacteriia*.

Based on the histograms of order (**Figure 6C**), *Lactobacillales*, belonging to *Bacilli*, accounted for 4.8% of the total amount of bacteria in the T group, which was higher than that in the C group (0.2%) and M group (1.2%) (*p*<0.01). *Actinomycetales* accounted for 0.007% and 0.006% of the total number of bacteria in the T and the M group, which were higher than that in the C group, which accounted for 0.002% (*p*<0.01). So, *Lactobacillales* was the specific bacteria in the T group, while *Actinomycetales* was the specific bacteria in the T and Mgroup.

In terms of the family histogram (**Figure 6D**), the abundance of *Prevotellaceae*, which was the specific bacteria in the C group, in the C group was higher than that in the other two groups (12.5% VS 5.1%, 3.5%) (*p*<0.01).

### 3.6 Comparison of Metastats differences between tumor without metastases and metastases group

**Figure 7:**
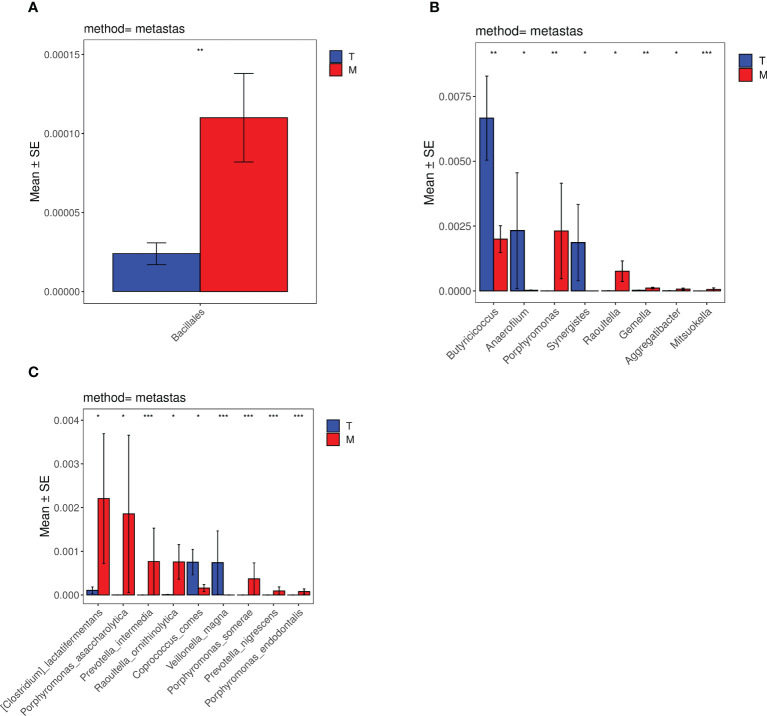
Histograms of distribution differences of species between the tumor without metastases group and the metastases group performed by Metastats software at the levels of order **(A)**, genus **(B)** and species **(C)**. *p < 0.05, **p < 0.01, ***p < 0.001, ****p < 0.0001.

The species differences between the T group and the M group were analyzed by metastats software. According to the histograms of order (**Figure 7A**), the differences in *Bacillales* between the T group and the M group were significant (0.002%<0.01%) (*p*<0.01). Based on the histograms of the genus (**Figure 7B**), the differences in *Mitsuokella* between the two groups were significant (0%<0.006%) (*p*<0.001). The differences of *Butyricicoccus*, *Gemella* and *Porphyromonas* were both significant (0.7%>0.2%,0.002%<0.01%,0.0002%<0.2%) (*p*<0.01). The differences of *Raoultella*, *Synergistes*, *Aggregatibacter* and *Anaerofilum* were statistically significant (0.0008%<0.08%,0.2%>0%,0.0009%<0.007%,0.2%>0.003%) (*p*<0.05). Considering the species histograms (**Figure 7C**), the differences of *Prevotella_intermedia*, *Veillonella_magna*, *Porphyromonas_somerae*, *Porphyromonas_endodontalis*, *Prevotella_nigrescens* were significant (0%<0.08%,0.08%>0%,0%<0.04%,0%<0.008%,0%<0.009%) (*p*<0.001). The differences of *[Clostridium]_lactatifermentans*, *Porphyromonas_asaccharolytica*, *Raoultella_ornithinolytica* and *Coprococcus_comes* between the two groups were statistically significant (0.01%<0.2%,0.0001%<0.2%, 0.0008%<0.08%,0.08%>0.02%) (*p*<0.05). However, the statistical differences in the histograms of family, class, and phylum between the experimental groups were not evident.

## 4 Discussion

Colorectal cancer is one of the common malignancies (17), and the survival of CRC patients is severely influenced by metastasis and recurrence (18). It turns out that the gut microbiota which is associated with the human normal metabolism state (19) plays a significant role in CRC formation and development (20). Yuan N et al. (21) demonstrated that alterations in gut microbiota composition could remodel the liver immune microenvironment by regulating Kupffer cells (KCs) on colorectal cancer, promoting or inhibiting liver metastases. Alice Bertocchi et al. (22) suggested that by breaking the intestinal vascular barrier and forming a premetastatic niche, the bacteria of primary colorectal cancer spread to the liver, thereby promoting the metastasis of colorectal cancer.

There is growing evidence that the gut microbiota diversity of patients with CRC decreased (23). Likewise, the results in our study showed that the diversity of microbial communities in the control group was higher than those in the tumor without metastases group and the metastases group. Comparing the taxonomic groups between healthy volunteers and patients in CRC, our study showed that *Bacteroidetes* was significantly more abundant in the control group than that in the CRC group, which was the most predominant phylum in healthy individuals. *Actinomycetales* and *Bacilli* were also enriched in the CRC group, while *Erysipelotrichia* and *Flavobacteriia* were less abundant in the CRC group. The changes and differences in the gut microbiota abundance between healthy people and CRC patients in our experiments were similar to the common experimental results at home and abroad (24–27), and a very small part of the difference may be caused by the influence of the treatment plan, relatively sample size and geographical factors like diet and climate.

Comparing the microbial communities between the tumor without metastases group and the metastases group, the results of our study showed the enrichment of *Fusobacterium*, *Porphyromonas*, *Raoultella*, *Lachnoanaerobaculum*, and *Ezakiella*, and the depletion of *Butyricicoccus* and *Succinatimonas* in CRC patients with metastases (*p*<0.05). In previous studies, the higher proportions of *Fusobacterium (* 28)*, Fusobacterium nucleatum (* 29), *Porphyromonas asaccharolytica*, and *Porphyromonas gingivalis (* 30), and the lower proportions of *Lachnospira multipara (* 31), *Prevotellaceae*, and *Butyricicoccus (* 32) were commonly observed in CRC patients with metastasis. In contrast, the differences observed in our study of *Fusobacterium*, *Porphyromonas*, and *Butyricicoccus* were similar to the previous results, while we also did detect meaningful differences in *Raoultella*, *Lachnoanaerobaculum*, *Ezakiella*, and *Succinatimonas*. In the tumor without metastases group, the predominant bacterium on the class level was *Bacilli*; on the family level was *Eubacteriaceae*; on the genus level was *Streptococcus, Butyricicoccus, Synergistes*, and *Anaerofilum*; on the species level was *Coprococcus_comes* and *Veillonella_magna*. The differences of them were statistically significant (*p*<0.05). Among our results, *Bacilli* and *Streptococcus* were the same as the former investigation (33–35), and we also obtained valid data of *Butyricicoccus*, *Veillonella_magna*, *Synergistes*, *Anaerofilum*, *Coprococcus_comes*, and *Eubacteriaceae*. Nevertheless, we did not detect meaningful data on *Escherichia*.

Gut microbiota can promote the proliferation and metastasis of tumor in multiple ways. *Porphyromonas gingivalis* is related to the occurrence and development of various of tumors (36–38). Mu W et al.  (39) found that *Porphyromonas gingivalis* could promote the proliferation of colorectal cancer cells by activating the MAPK/ERK signaling pathway. In addition, *Porphyromonas gingivalis* was found to promote the metastasis and malignant progression of lung cancer through long-term colonization of lung cancer cells  (40). *Porphyromonas* gingivalis and *Aggregatibacter* actinomycetemcomitans could initiate the Toll-like receptor (TLR) signaling pathways, and the activation of this pathway produced tumor-promoting effects (41, 42). *Raoultella* belongs to the *Enterobacteriaceae* family. The secretome of *Enterobacteriaceae* enhanced the growth of colorectal cancer cells (43). Besides, Rubinstein MR et al. (44) found that when the benign cells become cancerous and expressed elevated levels of Annexin A1, Fusobacterium nucleatum would be activated and stimulated the growth of colorectal cancer cells by the Wnt/ß‐catenin signaling. Our results showed enrichment of *Porphyromonas*, *Aggregatibacter*, *Raoultella*, and *Fusobacterium* in the metastases group, so we suggested that their enrichment may promote proliferation and metastasis of colorectal cancer cells and may serve as diagnostic markers for CRC progression.

Gut microbiota contribute to transformation and tumor progression by participating in metabolism and its metabolites. The most important metabolites in the gut microbiota are short-chain fatty acids (SCFAs), of which butyrate is an important member and has been found to have multiple beneficial effects on colon cancer (45). Hu S et al. (46) found that butyrate inhibited miR-92a transcription by reducing c-Myc, ultimately reducing cancer cell proliferation and stimulating apoptosis. Butyrate could also deactivate Akt/ERK signaling in histone deacetylase dependent manner, which impeded CRC cell metastasis and invasion (47). Chang S C et al. (48) found that *Butyricicoccus pullicaecorum*, a gut butyrate-producing bacterium, reduced the progression of 1,2-dimethylhydrazine-associated colorectal cancer by regulating short-chain fatty acid transporter and its receptor. *Butyricicoccus* could also downregulate the expression of PLAC8, which may be associated with CRC recurrence, and induce apoptosis in PLAC8-overexpressing cells (49). Therefore, we speculated that the reduction of *Butyricicoccus* is one of the diagnostic markers of colorectal cancer metastasis, and our experimental results showed that *Butyricicoccus* in CRC with metastasis was less abundant than that in CRC without metastasis, which supported this speculation. In addition, Ternes D et al. (50) found that formate, a metabolite of *Fusobacterium nucleatum*, drove CRC tumor invasion by triggering AhR signaling. Li R et al. (51) detected larger primary tumors, more liver metastatic foci, and higher LPS release in intestinal dysbiosis, which was brought about by the excessive administration of *Escherichia coli*. LPS increased the expression of CTSK in colorectal cancer cells, which promoted the invasion and metastasis of CRC cells by stimulating the secretion of cytokines by M2 TAMs and led to a poor prognosis. The abundance of *Prevotella*, *Mitsuokella*, and *Fusobacterium* was associated with Trimethylamine N-oxide (TMAO), a compound derived from diet and metabolism by the gut microbiome (52). TMAO is involved in a number of genetic pathways with an apparent association to carcinomas, especially colon cancer (53) and it has been found to exert oncogenic effects by promoting cell proliferation and angiogenesis in colorectal cancer (54).

Gut microbiota can alter the tumor microenvironment through inflammatory or immune responses and finally promote the occurrence and development of malignant tumor. *Aggregatibacter actinomycetemcomitans*, which was a rarely detected periodontopathic bacteria, was identified as being potentially associated with esophageal cancer, pancreatic cancer and precancerous gastric lesions (55). It produced several virulence factors such as cytolethal distending toxin (CDT) and leukotoxin (LtxA) to subvert the host immune response. CDT secreted from *Aggregatibacter* was pro-inflammatory and could promote carcinogenesis by creating a pro-inflammatory and or growth factor-rich microenvironment (56). LtxA could specifically target and kill activated white blood cells (WBCs) by binding to lymphocyte function-associated antigen-1 (LFA-1) (57). Therefore, *Aggregatibacter* may promote cancer progression by suppressing the host’s immune response, which was consistent with our finding of increased numbers in the metastatic group. Proença M A et al. (58) found that *Fusobacterium nucleatum* increased the expression of inflammatory mediators through possible miRNA-mediated activation of TLR2/TLR4. Therefore, the development of CRC was promoted through the immune responses to inflammatory stresses. In addition, Engevik MA et al. (59) found that outer membrane vesicles (OMVs) secreted from *Fusobacterium nucleatum* could promote proinflammatory cytokine production. Kostic A D et al. (60) also found that *Fusobacterium nucleatum* could increase tumor multiplicity and selectively recruited tumor-infiltrating myeloid cells in the mouse model, which generated a pro-inflammatory microenvironment and promoted CRC progression. *Porphyromonas gingivalis* promoted CRC’s formation and development (61) through inflammation of gut tissue (62). It plays a major role in the PD-L1 up-regulation in colon carcinoma cells, which could induce chronic inflammation and activate mechanisms of immune evasion (63). Our experimental results showed that *Aggregatibacter, Fusobacterium nucleatum*, and *Porphyromonas gingivalis* were the predominant bacteria in the metastases group, and combined with their above-mentioned functions and mechanisms, we suggested that this gut microbiota may serve as a supplement biological basis for the diagnosis of metastatic colorectal cancer.

Furthermore, gut microbes are closely related to the structure of the systemic immune system (64), the differences of intestinal microbiota in colorectal cancer patients before and after metastasis have influence on chemoradiotherapy and immunotherapy. *Eubacterium_limosum* which was the predominant flora in T group could enhance ICIs by inducing IFN-γ CD8 T cells (65). In the T group, the abundance of *Proteobacteria_eggerthii* was also higher than that in the M group, which was same with the result after CCRT that the abundance of *Proteobacteria* increased (66). After chemoradiotherapy, the *Bacteroidetes* abundance of individuals with short PFS was lower than that of individuals with long PFS, while the lower ratio may be associated with the progression and recurrence of colorectal cancer (67). Moreover, enrichment of *Firmicutes* could improve the sensitivity of ICI immunotherapy (68) and *Bacteroides* could enhance the anti‐CTLA‐4 therapy (69) and the anti-PD-1 immunotherapy (70). Clinically, it was also found that the abundance of *Bacteroides vulgatus* in anti-PD-1 blockade responders was higher than that in non-responders (64). *Bacteroides* played a role by upregulating systemic MDSCs and inducing Th1 mediated immune responses (71).The above results were consistent with our results. In our study, *Flavobacteriia* belonging to *Bacteroidetes* and *Erysipelotrichia* belonging to *Firmicutes* in the M group were lower than that in the T group. Therefore, according to our results combined with previous studies, we hypothesized that *Eubacterium_limosum, Proteobacteria, Bacteroidetes* and *Firmicutes* may optimize the effects of chemoradiotherapy and immunotherapy and slow the metastatic progression of colon cancer. Meanwhile Further imbalance of gut microbiota biodiversity can affect the treatment of therapy and promote the development of colorectal cancer. FOLFOX scheme is a chemotherapy regimen based on oxaliplatin and 5-FU which is one of the most commonly used chemotherapy regimens for patients diagnosed with metastatic colorectal cancer, whereas chemoresistance is the main problems in treatment (72). Study had shown that *Prevotella* and 3-Oxo not only promoted the development of malignant tumors, but also reversed the anticancer effect of FOLFOX (73). *Prevotella*, as an important carrier of drug resistance genes, was one of the dominant bacteria in the transfer group (67).

Consequently, by improving the structure of intestinal flora *in vivo*, colorectal cancer metastasis can be slowed down and the effects of radiotherapy, chemotherapy and immunotherapy can be enhanced. Spencer et al. (74) showed that modulation of gut microbes through dietary fiber and probiotics could enhance the effect of cancer immunotherapy. *Fusobacterium* was enriched in M group, but through the colonization of specific exogenous probiotics, its content in feces of patients could be reduced, thus promoting the effect of immunotherapy (75). On the contrary, *Lactobacillus_Mucosae* was also the dominant bacterium in M group, but study had shown that *Lactobacillus* could improve the efficiency of immunotherapy by enhancing PD-1/PD-L1 or CTLA-4 blockade (76). On the other hand, the use of immunotherapy to optimize the intestinal flora *in vivo* is also a breakthrough direction in the treatment of colorectal cancer in the future. It is generally believed that Treg/Th17 balance plays a fundamental role in stabilizing the homeostasis of intestinal microecology (77) and Treg cells participate in the development and progression of tumors by inhibiting anti-tumor immunity (78). TLR2/TLR4 can inhibit intestinal inflammation caused by Fusobacterium nucleatum *in vivo* by activating and inducing Tregs (79).

Despite the novel and meaningful findings, there are still some limitations in our study. Due to improper operation during collection, transportation, or storage, the sequencing data of the T16 was insufficient to reflect the vast majority of bacterial diversity information in the sample, and its analysis results were quite different from those in the same group. As for the differences in the abundance of *Bacteroidetes (* 27), *Firmicutes (* 32), and *Actinobacteria (* 34) between healthy people and CRC patients, there are few studies with opposite results. The occurrence of this phenomenon may be related to the geographic location, diet habits, and age characteristics of the patients, the activity, the extent and treatment of the CRC, and the difference in DNA extraction methods. Therefore, larger sample sizes, more groups, and further experimental studies are still required.

The microbiome has received extensive attention in recent years. In the annual report of ASCO (80, 81), the work of identifying biomarkers relevant to immunotherapies that predicted initial response, long-term disease control, adverse events, resistance, and the microenvironment of potentially malignant lesions that were associated with progression to invasive disease were recognized as priority focus areas. And in the 2021 annual meeting special issue of CSCO, gut microbiota was thought of as a predictive biomarker for the occurrence and development of irAEs. It was expected to reverse the destruction of intestinal flora homeostasis and blocked its impact on the human body through a series of methods such as intestinal flora transplantation, probiotic intervention, and targeted drugs for specific intestinal flora. Although there have been lots of studies on the relationship between the gut microbiome and colorectal cancer, studies on the association of gut microbiome with colorectal cancer metastasis are still limited. Therefore, our study innovatively discovered the difference among healthy people, patients in colorectal cancer with and without metastases, which leads to the exploration of the pathogenesis and development of colorectal cancer from the perspective of gut microbiota, contributing to understanding the tumorigenesis of colorectal cancer from microbiological perspective. We hope that this study may offer some novel perspective for the precise treatment of CRC by targeting specific microbiota, which may help to realize the personalized colorectal cancer treatment mediated by the gut microbiota.

## 5 Conclusion

In summary, our study suggests the decreased diversity of gut microbiota in CRC patients and the differences in composition, abundance and predominant bacteria of flora between the non-metastatic patients and metastatic patients. *Synergistes*, *Anaerofilum*, *Coprococcus_comes*, *Eubacteriaceae*, *Bacilli*, *Streptococcus*, *Butyricicoccus*, and *Veillonella_magna* are the predominant bacteria in the tumor without metastases group, while *Fusobacteria*, *Porphyromonas*, *Prevotella*, *Mitsuokella*, *Gemella*, *Raoultella*, *Aggregatibacter*, and *[Clostridium]_lactatifermentans* are the predominant bacteria in the metastases group. Therefore, gut microbiome analysis may offer the potential to develop non-invasive diagnostic tests, serve as a supplement biological basis for the diagnosis and treatment of metastatic colorectal cancer and improve the effectiveness of treatment.

## Data availability statement

All the protocols and procedures of this study were approved by the Zhejiang Province Hospital of TCM Ethics Committee (2020-KL-050-01), and all volunteers signed the informed consent form before participating in the experiment.

## Author contributions

LS, ZZ, XJ: These authors have contributed equally to this work and share first authorship. PW, SZ, JY: These authors are responsible for corresponding. All authors contributed to the article and approved the submitted version.

## Funding

This study was supported by Natural Science Foundation of Zhejiang Province (LQ22H270008); China Postdoctoral Science Foundation (2021M702928); The Postgraduate Scientific Research Fund of Zhejiang Chinese Medical University (2021YKJ01); Young Elite Scientists Sponsorship Program by CACM (2021-QNRC2-B13).

## Conflict of interest

The authors declare that the research was conducted in the absence of any commercial or financial relationships that could be construed as a potential conflict of interest.

## Publisher’s note

All claims expressed in this article are solely those of the authors and do not necessarily represent those of their affiliated organizations, or those of the publisher, the editors and the reviewers. Any product that may be evaluated in this article, or claim that may be made by its manufacturer, is not guaranteed or endorsed by the publisher.
